# Electromyography biofeedback therapy for spinal cord injury rehabilitation: a systematic review

**DOI:** 10.3389/fneur.2026.1885605

**Published:** 2026-10-13

**Authors:** Ming-wei Liu, Zhong-yuan Liu, Bing-can Zhang, Shu-ji Gao, Hui-de Peng

**Affiliations:** 1 Department of Emergency, Dali Bai Autonomous Prefecture People’s Hospital, Dali, Yunnan, China; 2 Department of Nursing, Dali Bai Autonomous Prefecture People’s Hospital, Dali, Yunnan, China; 3 Department of Spine Surgery, Dali Bai Autonomous Prefecture People’s Hospital, Dali, Yunnan, China; 4 Department of Emergency, The First Hospital Affiliated with Kunming Medical University, Kunming, Yunnan, China; 5 Department of Medical Administration, Dali Bai Autonomous Prefecture People’s Hospital, Dali, Yunnan, China

**Keywords:** electromyography biofeedback therapy, rehabilitation, spinal cord injury, surface electromyography, systematic review

## Abstract

**Background:**

Electromyographic biofeedback (EMG-BF) is being increasingly used as an adjunct to rehabilitation after spinal cord injury (SCI), but the available evidence is limited by the small sample size, heterogeneous comparators, and variable outcome reporting. We aimed to systematically identify and narratively synthesize the evidence on the effects of EMG-BF in SCI rehabilitation.

**Methods:**

We searched the PubMed, Embase, Cochrane Library, Web of Science, CNKI, Wanfang, VIP and other databases from inception to 8 January 2026. Two reviewers independently screened records, extracted data and assessed the risk of bias using the Cochrane RoB 2 tool. Because of substantial clinical and methodological heterogeneity and inconsistent outcome-data types, we did not pool effect estimates; instead, we performed a structured synthesis without meta-analysis (SWiM) and graded the certainty of evidence using GRADE.

**Results:**

Ten randomized controlled trials (RCTs) involving 537 participants were included. All 10 studies reported a favorable direction of effect for EMG-BF, but the evidence for every outcome was of low or very low certainty. Across the five RCTs reporting surface electromyography (sEMG), all reported larger signal amplitudes in the EMG-BF arm, although the outcome data types varied (means vs. medians) and the muscle sites and comparators differed. Two RCTs reported greater lower-limb muscle strength with EMG-BF. One RCT reported a higher functional independence measure score, and two reported higher ASIA motor scores with the use of the EMG-BF. Four RCTs reported reduced spasticity (modified Ashworth scale, MAS), but MAS was reported inconsistently as ordinal grade distributions or as grade-derived scores, precluding any pooled estimate. Three RCTs reported higher Berg balance scale scores with the use of the EMG-BF. Only one pilot RCT reported pain, and it permitted only within-group comparisons.

**Conclusion:**

Low- to very-low-certainty evidence suggests that EMG-BF may improve sEMG signals, lower-limb muscle strength, functional independence, ASIA motor scores, spasticity and balance in patients with SCI, but these findings are preliminary. Large, well-designed RCTs with allocation concealment, blinding of outcome assessors, standardized comparators and prospective registration are needed.

**Systematic review registration:**

https://www.crd.york.ac.uk/PROSPERO/view/CRD420261282923, Identifier: [CRD420261282923].

## Highlights

- Spinal cord injury (SCI) often leads to partial or complete loss of function below the level of injury, significantly impacting quality of life.
- Electromyographic biofeedback (EMG-BF) can improve specific clinical outcomes following SCI.
- Because of heterogeneous comparators and inconsistent outcome data types, we synthesized the evidence narratively (SWiM) rather than by meta-analysis.
- Low- to very-low-certainty evidence suggests that the EMG-BF may improve sEMG signals, muscle strength, functional independence, ASIA motor scores, spasticity and balance in patients with SCI.

## 1 Introduction

Spinal cord injury (SCI) is a neurological disorder that affects 250,000 to 500,000 individuals globally each year and often leads to partial or complete loss of function below the level of injury, significantly impacting quality of life (1). The restoration of walking ability is a primary concern for many patients. Prognosis for walking function is primarily associated with injury severity (ASIA Impairment Scale [AIS] classification) and the neurological level of injury, together with the preservation of descending motor pathways, residual voluntary muscle strength and sensorimotor integrity (2, 3). Electromyography biofeedback therapy (EMG-BF) is an active, conscious rehabilitation technique that provides patients with real-time feedback of muscle electrical activity, usually in visual or auditory form, to promote muscle contraction and facilitate motor relearning (4).

Previous studies have shown that electromyographic biofeedback electrical stimulation (EMG-BFES) can improve specific clinical outcomes following SCI, including muscle activation, muscle strength, spasticity scores, selected functional performance measures and pain scores (5, 6). Functional impairment after SCI is multifaceted, and the outcomes examined in this review pertain primarily to motor and functional domains, together with spasticity and pain. However, few small-sample, single-center trials (sample sizes < 50) and multicenter RCTs have been conducted to validate the long-term efficacy of these treatments. This study aims to systematically integrate existing research and provide a structured narrative synthesis of the efficacy of EMG-BF in the rehabilitation of patients with SCI, with the goal of providing evidence and guidance for future clinical practice and research.

## 2 Materials and methods

### 2.1 Search strategy

Comprehensive computerized searches were conducted in PubMed, Embase, Web of Science, the Cochrane Library, CNKI, Wanfang, and the VIP Chinese Science and Technology Journal Database from the inception of each database to 8 January 2026. Both Chinese and English search terms were used, and the full search strategies for each database are provided in the Supplementary materials. The reference lists of the included studies were also screened to identify additional relevant studies.

### 2.2 Inclusion and exclusion criteria

Studies were included if they met all of the following criteria: (1) a randomized controlled trial (RCT) design; (2) participants with a clinical diagnosis of SCI; (3) EMG biofeedback as the experimental intervention, alone or added to conventional rehabilitation; (4) at least one outcome within the review scope was reported; and (5) the full text was available.

Studies were excluded if they met any of the following criteria: (1) nonrandomized study designs (including quasiexperimental, time-sequential or historical-control designs and crossover trials that did not evaluate EMG-BF against a control intervention); (2) reviews, case reports, conference abstracts or other nonprimary research; (3) duplicate publications of the same patient cohort; and (4) studies whose outcomes fell outside the scope of the review. Study eligibility was determined strictly according to these predefined criteria and was not influenced by the effect of any individual study on the results.

### 2.3 Data extraction

Two researchers independently screened the literature, extracted the data and crosschecked the findings, with disagreements resolved through discussion or third-party consultation. The data extracted included (1) basic information such as the first author, publication year and sample size; (2) patient characteristics such as age, sex, injury severity, injury location and duration of illness; and (3) intervention details, including the EMG-BF protocol (frequency, intensity, duration) and conventional rehabilitation content. Every extracted value was recorded together with its source location in the primary report so that each number in the synthesis was traceable to its source.

### 2.4 Outcome measures

The outcomes of interest were surface electromyography (sEMG) of the lower-limb muscles, manual muscle testing of lower-limb muscle strength (quadriceps, hamstrings and tibialis anterior), the Functional Independence Measure (FIM), the American Spinal Injury Association (ASIA) motor score, the Modified Ashworth Scale (MAS), the Berg Balance Scale (BBS) and validated pain rating scales. All outcome measures are instruments validated for, and routinely applied in, the SCI population.

### 2.5 Risk of bias assessment

The risk of bias of the included randomized controlled trials was assessed using the revised Cochrane risk-of-bias tool for randomized trials (RoB 2) (7), which evaluates five domains: bias arising from the randomization process; bias due to deviations from the intended interventions; bias due to missing outcome data; bias in measurement of the outcome; and bias in selection of the reported result. Two reviewers independently assessed each study, and disagreements were resolved through discussion or consultation with a third reviewer. The results are presented as a traffic-light plot (Figure 1) and in Supplementary Table S1, which records the domain-level judgments and their supporting quotations for each study.

**Figure 1:**
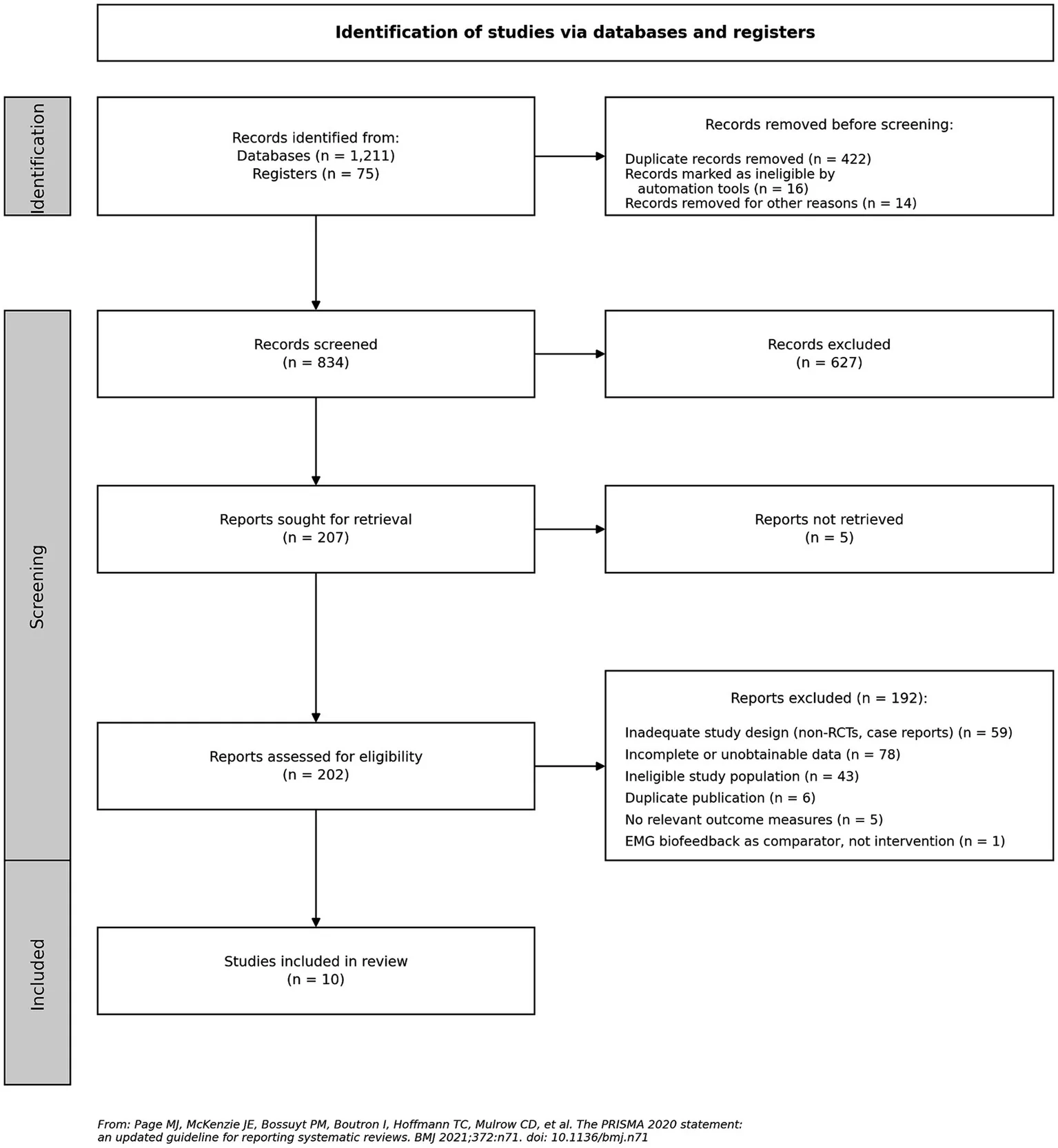
PRISMA 2020 flow diagram of the study selection process. A total of 1,286 records were identified from databases (*n* = 1,211) and registers (*n* = 75). After 452 records were removed before screening (422 duplicates, 16 records marked as ineligible by automation tools, and 14 records removed for other reasons), 834 records underwent title and abstract screening, of which 627 were excluded. The full text of 207 reports was sought for retrieval; 5 reports could not be retrieved, and 202 reports were assessed for eligibility, of which 192 were excluded and 10 randomized controlled trials were included in the review.

### 2.6 Synthesis methods

Quantitative pooling was judged to be inappropriate after complete re-extraction and verification of the primary data. The reasons were (1) marked clinical heterogeneity in study populations, outcome definitions and intervention protocols; (2) methodological heterogeneity in the comparators, which ranged from conventional rehabilitation alone to several different active alternatives; and (3) inconsistent outcome-data types, most notably the Modified Ashworth Scale, which was reported as ordinal grade distributions in some studies and as grade-derived numeric scores in others, and sEMG and muscle strength, which were reported as medians (interquartile ranges) in one study and as the means (SD) in others. We therefore performed a structured synthesis without meta-analysis, reported according to the Synthesis Without Meta-analysis (SWiM) guidelines (8).

Within each outcome, studies were grouped by comparator type (conventional rehabilitation versus active comparator), and for each study, we reported the direction of effect (whether EMG-BF was favored), whether the difference was reported as statistically significant, the outcome-data type, and the risk-of-bias context. We did not compute or report any pooled effect estimates, and no forest plots are presented.

### 2.7 Certainty of evidence

The certainty of the evidence for each outcome was assessed using the Grading of Recommendations Assessment, Development and Evaluation (GRADE) approach (9, 10). Evidence from randomized trials was initially rated as high certainty and was downgraded by one or two levels for each of five domains: risk of bias, inconsistency, indirectness, imprecision and publication bias. Two reviewers independently applied the GRADE criteria, and disagreements were resolved by discussion. Because no pooled estimates were computed, the summary-of-findings table (Table 1) reports the number of studies and participants, the certainty rating, and a narrative summary of the direction and consistency of findings, rather than an effect size.

**Table 1:** Basic characteristics of the included studies (*n* = 10).

Author and year	Study design	Research subjects	Sample size (E/C)	Experimental group	Control group	Outcome indicator
He et al. 2011 (11)	RCT (random number table)	Patients with spinal cord injury	35/35	Electromyographic biofeedback training added to the control group	Conventional rehabilitation + medium-frequency electrotherapy (active)	① sEMG; ④ ASIA motor score
Middaugh et al. 2013 (12)	RCT (pilot)	SCI, C6 or below, manual wheelchair users, shoulder pain	8/7	EMG biofeedback added to exercise	Home-based standard exercise programme	⑦ Pain (within-group only)
Govil and Noohu (2013) (13)	RCT (pretest–posttest)	Incomplete SCI, WISCI ≥6, 18–50 y, ≥1 y post-injury	30 (per-group *n* not reported)	Gluteus maximus EMG biofeedback added to conventional rehab + gait training	Conventional rehabilitation + gait training	① sEMG
Shi et al. (2014) (14)	RCT	SCI after surgery, ankle dorsiflexion dysfunction	15/15	EMG biofeedback electrical stimulation added to the control group	Ankle-foot orthosis (90°) + conventional rehabilitation	① sEMG; ② lower-limb muscle strength‡
Hu et al. (2016) (15)	RCT	Incomplete thoracolumbar SCI	30/30	EMG biofeedback added to conventional rehabilitation	Conventional rehabilitation	① sEMG; ③ FIM; ④ ASIA motor score
Xie and Ma (2019) (16)	RCT (random number table)	SCI with lower-limb spasticity	27/27	EMG biofeedback added to conventional rehabilitation	Conventional rehabilitation	⑤ MAS (ordinal *n*(%))
Gong et al. (2021) (17)	RCT (three-arm)	SCI with lower-limb spasticity	40/40/40 (A/B/C)	A: EMG biofeedback + suspension exercise; B: EMG biofeedback	C: suspension exercise (active)	⑤ MAS; ⑥ BBS; FAC; CSS; Fugl-Meyer
Guo et al. (2021) (18)	RCT (block randomisation)	Incomplete paraplegia, quadriceps < grade 3, >6 mo post-injury, <60 y	40 randomised (33 analysed)	EMG biofeedback added to conventional physiotherapy	Conventional physiotherapy	① sEMG (median, IQR)
Yang et al. (2023) (19)	RCT (random number table)	Patients with spinal cord injury	21/21	EMG biofeedback electrical stimulation (incl. Pelvic-floor stimulation) added to conventional rehab	Conventional rehabilitation	⑤ MAS; ⑥ BBS; LMES; 10MWT
Chen et al. (2024) (20)	RCT (random number table)	Patients with spinal cord injury	38/38	EMG biofeedback added to core-stability training	Core-stability training on unstable surfaces (active)	⑤ MAS; ⑥ BBS; FAC; FMA; BI

① Surface electromyography (sEMG); ② lower limb muscle strength; ③ Functional Independence Measure (FIM); ④ ASIA Motor Scale; ⑤ Modified Ashworth Scale (MAS); ⑥ Berg Balance Scale (BBS); ⑦ pain score. Active comparators are marked in bold. All included studies were randomized controlled trials (RCTs).

Active comparators are marked in bold. Govil and Noohu (13) reported a total of 30 participants but did not state the number per group. Guo et al. (18) randomised 40 participants and analysed 33; the per-outcome participant counts in Table 1 use the analysed number. ‡Shi et al. (14) reported a muscle-strength value (1.04 ± 1.38 → 4.20 ± 0.82) that is implausible for the 0–5 grade scale and is identical to the tibialis-anterior sEMG value in Hu et al. (15); this is flagged as a data concern in the synthesis.

## 3 Results

### 3.1 Literature search results

A systematic search of PubMed, Embase, the Cochrane Library, Web of Science, CNKI, Wanfang and VIP from database inception to 8 January 2026 identified 1,286 records (1,211 from databases and 75 from registers). After removing 452 records before screening (422 duplicates, 16 records marked as ineligible by automation tools, and 14 records removed for other reasons), 834 records underwent title and abstract screening, of which 627 were excluded. The full text of 207 reports was sought for retrieval; 5 reports could not be retrieved, and 202 reports were assessed for eligibility. Of these 202 reports, 192 were excluded and 10 randomized controlled trials (11–20) were included in the review. The 192 exclusions comprised 59 reports with inadequate study design (non-RCTs, case reports and other non-primary designs), 78 with incomplete or unobtainable data, 43 with an ineligible study population, 6 duplicate publications, 5 without relevant outcome measures, and 1 in which EMG biofeedback was the comparator rather than the intervention. Six of these exclusions were made during the present revision after re-application of the prespecified criteria: three non-randomized trials (21–23), one in which EMG biofeedback was the comparator rather than the intervention (24), one whose outcomes were outside the review’s scope (25), and one duplicate report of the same cohort as an included study (26). The study selection process is presented in the PRISMA 2020 flow diagram (Figure 2).

**Figure 2:**
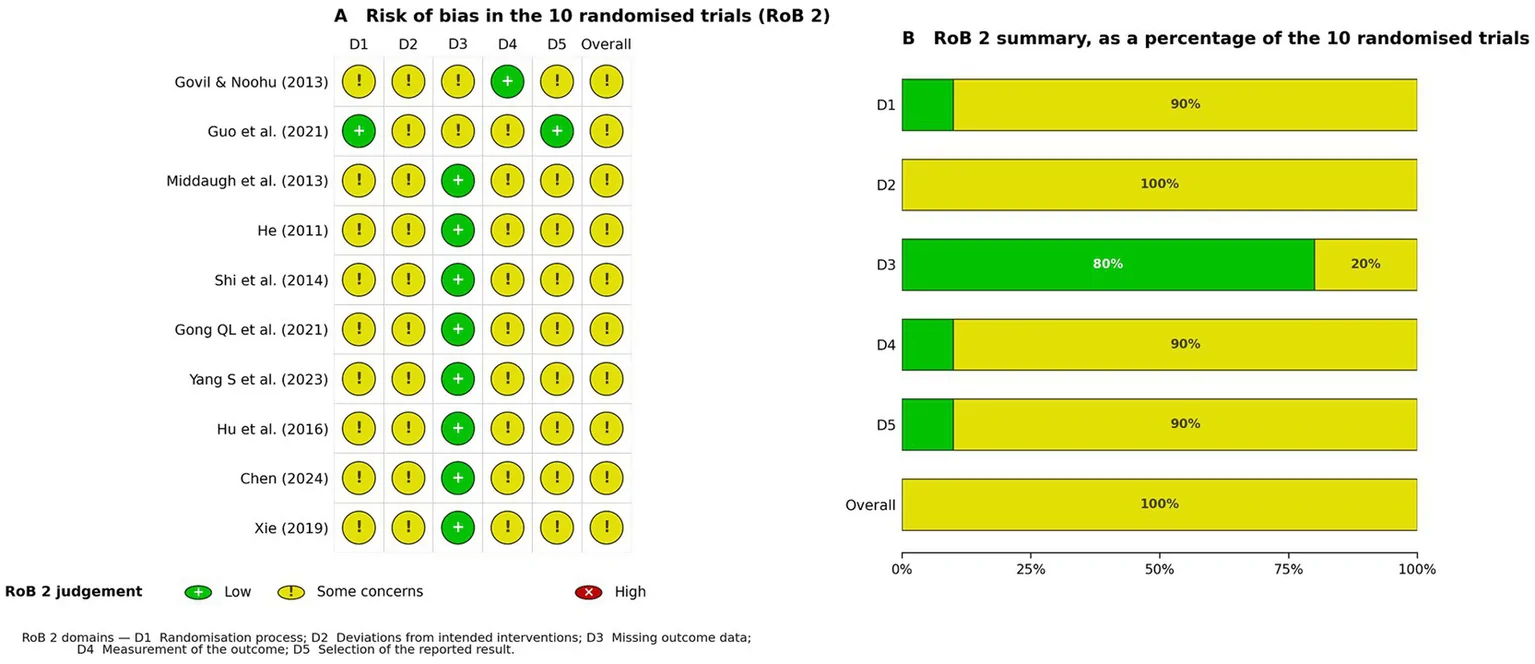
Risk of bias in the included studies. **(A)** Traffic-light plot of the domain-level and overall judgments for the 10 randomized controlled trials, assessed with the Cochrane risk-of-bias tool for randomized trials (RoB 2). **(B)** RoB 2 judgments expressed as a percentage of the 10 randomized controlled trials. The green “+” denotes a low risk of bias, the yellow “!” represents some concerns (RoB 2), and the red “×” represents high (RoB 2).

### 3.2 Basic characteristics of the included studies

As detailed in Table 2, the 10 included RCTs included patients with SCI, with sample sizes ranging from 15 to 120 participants. In every included study, the experimental group received EMG biofeedback, alone or in combination with the control intervention. The comparator conditions were heterogeneous and fell into two broad groups:

- Conventional rehabilitation (usual-care comparator): Hu et al. (15), Yang and Wu (19), Xie and Ma (16), Shi et al. (14), Guo et al. (18), Govil and Noohu (13), and Middaugh et al. (12).
- Active comparators: He et al. (11), Chen and Zhang (20), and Gong and Zhang (17).

**Table 2:** Summary of findings: electromyography biofeedback (EMG-BF) added to rehabilitation for functional outcomes after spinal cord injury (structured synthesis without meta-analysis).

Outcome	Studies (participants)	Certainty of evidence (GRADE)	Narrative summary of findings
Surface electromyography (sEMG)	5 (223)	⊕⊝⊝⊝ Very low	All 5 RCTs reported larger sEMG signal in the EMG-BF arm than in control; direction was consistent, but studies pooled heterogeneous muscle sites, several used different comparators, one reported median (IQR), and all were at some concern of bias. Downgraded for risk of bias, indirectness and imprecision.
Lower-limb muscle strength	2 (63)	⊕⊝⊝⊝ Very low	Two RCTs reported greater strength with EMG-BF; one reported median (IQR), and one reported an implausible grade-scale value identical to another study’s sEMG value. Downgraded for risk of bias, indirectness and imprecision.
Functional Independence Measure (FIM)	1 (60)	⊕⊝⊝⊝ Very low	A single RCT reported a higher FIM score with EMG-BF; the estimate is from one small study at some concern of bias and is imprecise. Downgraded for risk of bias and imprecision.
ASIA motor score	2 (130)	⊕ ⊕ ⊝⊝ Low	Two RCTs reported a higher ASIA motor score with EMG-BF; direction consistent but based on two small studies at some concern of bias. Downgraded for risk of bias.
Modified Ashworth Scale (MAS)	4 (292)	⊕⊝⊝⊝ Very low	MAS is ordinal; two RCTs reported grade distributions *n*(%), two reported grade-derived scores as mean ± SD. All reported reduced spasticity with EMG-BF, but the inconsistent data type precludes any pooled estimate. Downgraded for risk of bias, indirectness and imprecision.
Berg Balance Scale (BBS)	3 (238)	⊕ ⊕ ⊝⊝ Low	Three RCTs reported higher balance scores with EMG-BF; direction consistent but small samples at some concern of bias. Downgraded for risk of bias and imprecision.
Pain	1 (15)	⊕⊝⊝⊝ Very low	A single pilot RCT reported only within-group comparisons; no between-group effect could be estimated. Downgraded for risk of bias, indirectness and imprecision.

Population: adults with spinal cord injury. Intervention: EMG biofeedback added to rehabilitation. Comparison: conventional rehabilitation or another non-EMG-BF control. Because of clinical and methodological heterogeneity and inconsistent outcome-data types, outcomes are synthesised narratively (SWiM); no pooled effect estimates are reported.

GRADE certainty of evidence: ⊕ ⊕ ⊕ ⊕ High; ⊕ ⊕ ⊕⊝ Moderate; ⊕ ⊕ ⊝⊝ Low; ⊕⊝⊝⊝ Very low. Reasons for downgrading: risk of bias (all studies at ‘some concerns’ on RoB 2, with allocation concealment and blinding unreported in almost all studies); indirectness (sEMG and muscle strength are surrogate body-function measures, not patient-important outcomes); imprecision (small samples below the optimal information size). Publication bias was not formally assessed because every outcome included fewer than 10 studies. ASIA, American Spinal Injury Association; BBS, Berg Balance Scale; FIM, Functional Independence Measure; MAS, Modified Ashworth Scale; RoB 2, Cochrane risk-of-bias tool for randomised trials, version 2; sEMG, surface electromyography; SWiM, Synthesis Without Meta-analysis.

This heterogeneity of the comparator is examined in the Discussion section. The outcome measures included (1) sEMG, (2) lower-limb muscle strength, (3) FIM, (4) ASIA motor score, (5) MAS for spasticity, (6) BBS, and (7) pain. Adverse events were not reported consistently across the included studies; therefore, this review makes no claim regarding the safety of the EMG-BF.

### 3.3 Risk of bias

The risk of bias was independently assessed by two reviewers. All 10 included studies were randomized controlled trials and were assessed with the Cochrane RoB 2 tool. None of the 10 RCTs were judged to be at low risk of bias overall: all 10 were rated as having some concerns, and no RCT was rated at high risk in any domain (Figure 1; Supplementary Table S1).

*Randomization process (D1)*: Of the 10 RCTs, eight described a specific method for generating the random allocation sequence (random number table, computer-generated sequence, or block randomization), whereas two stated only that participants were randomized without describing how. Only one study, Guo et al. (18), was judged at low risk for this domain because it described block randomization performed by a specialist who was not involved in the intervention or assessments, together with consecutively numbered sealed envelopes for allocation concealment and balanced baseline characteristics. The remaining nine RCTs were judged as having some concerns, predominantly because allocation concealment was not reported; a baseline imbalance in ASIA impairment grade between groups was noted in Govil and Noohu (13).

*Deviations from intended interventions (D2)*: All 10 RCTs were rated as having some concerns. The interventions were behavioral and physical rehabilitation programmes in which participants and personnel cannot readily be blinded, and no study reported the blinding of participants or personnel.

*Missing outcome data (D3)*: Eight RCTs were judged to be low risk because the outcome data were reported for the same number of participants as were randomized, and no losses were described. Two RCTs were rated as having some concerns: Guo et al. (18) recruited 40 participants but analyzed only 33 participants without explaining the 7 missing participants, and Govil and Noohu (13) did not clearly report the number per group.

*Measurement of the outcome (D4)*: One RCT (Govil and Noohu, (13)) was judged at low risk because its outcomes were objective, instrument-measured quantities. The remaining nine RCTs were rated as having some concerns because the outcome was subjective or semisubjective (pain rating scales, MAS, BBS, FIM, or manual muscle-grading scales), and blinding of the outcome assessor was not reported.

*Selection of the reported result (D5)*: Only Guo et al. (18) was judged to be at low risk because the trial was prospectively registered (ChiCTR1800018891). The other nine RCTs were rated as having some concerns because they reported no prospective registration or published protocol.

In summary, all 10 included studies carried at least some concerns of bias, and no study was at a low risk of bias overall. This limits the certainty of the evidence, as reflected in the results of the GRADE assessment (Table 1).

### 3.4 Surface electromyography (sEMG)

Five RCTs reported sEMG outcomes (He et al. (11); Govil and Noohu (13); Shi et al. (14); Hu et al. (15); Guo et al. (18)). All five reported a larger sEMG signal in the EMG-BF arm than in the control arm; thus, the direction of the effect was consistent across studies. However, the studies differed in the muscle sites measured (gluteus maximus, quadriceps femoris, and tibialis anterior), in the comparator used, and in the outcome-data type: four studies reported means (SD), whereas Guo et al. (18) reported medians (25th, 75th percentiles), which could not be combined with the mean-based results. Two studies warrant particular caution: Govil and Noohu (13) reported internally inconsistent cadence values across their tables and did not report the number of participants per group, and He et al. (11) used an active comparator (medium-frequency electrotherapy) rather than conventional rehabilitation alone. For example, He et al. (2011) (11) reported that the quadriceps femoris sEMG signal increased from 65.9 ± 11.8 μV to 131.5 ± 19.3 μV in the EMG-BF group, whereas it increased from 72.6 ± 14.3 μV to 84.3 ± 19.1 μV in the medium-frequency electrotherapy control group (*p* < 0.05). Taken together, the sEMG evidence consistently favors the EMG-BF but is of very low certainty.

### 3.5 Lower-limb muscle strength

Two RCTs reported lower limb muscle strength (Shi et al. (14); Guo et al. (18)). Both reported greater muscle strength with EMG-BF. Guo et al. (18) reported the median (IQR) muscle strength. Shi et al. (14) reported that Lovett-graded muscle strength improved from 1.04 ± 1.38 to 4.20 ± 0.82 in the EMG-BF group versus 1.64 ± 1.47 to 3.10 ± 0.40 in the control group; this value should be interpreted cautiously because it is implausible for a 0–5 grade scale and is identical to the tibialis-anterior sEMG value reported in Hu et al. (15), raising a concern of mislabeling. The evidence of muscle strength is therefore very low certainty.

### 3.6 Functional Independence measure (FIM)

A single RCT (Hu et al. (15)) reported FIM, with the EMG-BF group improving from 54.82 ± 8.35 to 92.07 ± 9.25 (8 weeks) and 109.5 ± 13.82 (12 weeks). This is a single small study with some concern of bias, and the FIM evidence has very low certainty.

### 3.7 ASIA motor score

Two RCTs reported ASIA motor scores (Hu et al. (15); He et al. (11)). Both reported higher ASIA motor scores with the use of EMG-BF. Hu et al. (15) reported that the ASIA motor score improved from 11.48 ± 7.08 to 28.12 ± 11.28 (8 weeks) and 39.76 ± 13.45 (12 weeks) in the EMG-BF group. He et al. (11) reported that the ASIA motor score improved from 26.75 ± 5.07 to 32.65 ± 5.22 in the EMG-BF group versus 25.10 ± 4.26 to 28.50 ± 4.46 in the medium-frequency electrotherapy control group (*p* < 0.01 between groups). The two studies were consistent in direction but small and at some level of concern of bias, indicating low certainty of the evidence. He et al. (11) reported a psychological outcome (Rosenberg Self-Esteem Scale, RSES) that falls outside the motor and functional scope of this review; it improved in the EMG-BF group (18.3 ± 1.7 to 20.6 ± 2.5, *p* < 0.05) but not in the control group and correlated with the ASIA motor score in the EMG-BF group (*r* = 0.692, *p* < 0.01).

### 3.8 Modified Ashworth scale (MAS)

Four RCTs reported MAS for spasticity (Xie and Ma (16); Gong and Zhang (17); Yang and Wu (19); Chen and Zhang (20)). All four studies reported reduced spasticity with EMG-BF, so the direction of the effect was consistent. Critically, however, MAS has been reported inconsistently across studies: Xie and Ma (16) and Chen and Zhang (20) reported ordinal grade distributions (*n*, %), whereas Gong and Zhang (17) and Yang and Wu (19) converted grades to numeric scores and reported means (SD). Because MAS is an ordinal scale, none of these reports are suitable for combining into a continuous mean difference, and no pooled estimate was computed. Notably, in the three-arm trial by Gong and Zhang (17) in 2021, the MAS reduction was greater in the combined EMG-BF-plus-suspension arm (3.65 ± 0.34 → 2.15 ± 0.35) than in the suspension-only arm (3.60 ± 0.31 → 3.00 ± 0.20), indicating that compared with suspension exercise alone, adding EMG-BF to suspension exercise resulted in a greater reduction in spasticity. Because the comparator was itself an active intervention, these findings support an additional benefit of EMG-BF relative to an active alternative for spasticity. The MAS evidence is very low certainty.

### 3.9 Berg balance scale (BBS)

Three RCTs reported BBS (Gong and Zhang (17); Yang and Wu (19); Chen and Zhang (20)). All three reported higher balance scores with the use of the EMG-BF. In Gong and Zhang (17), BBS improved more in the EMG-BF-plus-suspension arm (35.65 ± 3.45 → 46.75 ± 4.28) than in the suspension-only arm (35.80 ± 3.28 → 41.60 ± 3.84); however, the point estimate for the EMG-BF-only arm versus the suspension arm was near zero, indicating little additional balance benefit of the EMG-BF over an active alternative. Yang and Wu (19) and Chen and Zhang (20) reported higher BBS in the EMG-BF arm than in the control arm did. The BBS evidence is low certainty.

### 3.10 Pain

A single pilot RCT (Middaugh et al. (12), *n* = 15) reported pain using the Wheelchair Users Shoulder Pain Index, but the authors reported only within-group comparisons (“allowed only within-group comparisons”); thus, no between-group effect of EMG-BF on pain could be estimated. The evidence of pain is very low certainty.

### 3.11 Certainty of evidence

The certainty of the evidence was graded as low or very low for all seven outcomes; no outcome reached moderate or high certainty (Table 1). The main reasons for downgrading were consistent across outcomes:

- Risk of bias: All 10 RCTs (11–20) were at some concern of bias, with allocation concealment and blinding unreported in almost all the studies.
- Indirectness: sEMG and muscle strength are surrogate body function measures rather than patient-important outcomes, and several comparators were active interventions rather than conventional rehabilitation.
- Imprecision: Every outcome was based on one to five small studies, with total sample sizes well below the optimal information size.

Publication bias was not formally assessed because every outcome included fewer than 10 studies, a threshold below which funnel-plot asymmetry and regression tests are unreliable. Taken together, the available evidence provides only low to very low certainty that electromyography biofeedback therapy improves the reported outcomes, and this should be borne in mind when interpreting the results.

## 4 Discussion

It was once believed that the central nervous system (CNS) has minimal regenerative capacity (27). However, recent studies have demonstrated that connections between CNS cells undergo continuous modification and refinement throughout life and that latent pathways and synapses can act as reserves that may be activated through targeted training (27). Axonal sprouting and growth are generally more pronounced in incomplete SCI (28). Clinical studies suggest that EMG-BF may enhance CNS plasticity and promote neural synapse regeneration (29–31), although much of this mechanistic evidence is derived from preclinical models and small clinical series, and its therapeutic relevance is clearest for individuals with incomplete injury who retain some descending motor pathways. The present review was conducted to integrate the available clinical evidence on the effects of EMG-BF in patients with SCI.

EMG-BF has been increasingly used for functional rehabilitation after SCI (18). As early as 2001, Petrofsky (32) highlighted EMG-BF as a rehabilitation technique for neurological disorders, including SCI. The clinical evidence, however, is mixed and often derived from small studies. For example, in a randomized trial excluded from the present review because EMG biofeedback was the comparator rather than the intervention (24), EMG biofeedback relaxation training and self-hypnosis training produced similar reductions in chronic pain, and a statistically significant reduction was observed in the self-hypnosis arm rather than the biofeedback arm—a finding that cautions against attributing benefit to biofeedback beyond the effects of an active comparator (33, 34). Jiang et al. (35) reported that EMG-BF improves neurogenic bladder function in patients with SCI; De Biase et al. (39) reported an enhanced voluntary response of the rectus femoris; Yang and Wu (19) reported that EMG-BF electrical stimulation improves lower-limb function and walking ability; and Govil and Noohu (13) reported that adding gluteus maximus EMG-BF to conventional rehabilitation improved gait parameters. In the present synthesis, all included studies reported a favorable direction of effect for EMG-BF across the motor, functional, spasticity and balance outcomes examined.

The findings for the individual outcomes are nonetheless preliminary. With respect to sEMG, all five contributing studies (11, 13–15, 18) reported larger signal amplitudes with EMG-BF, but the studies pooled heterogeneous muscle sites and used different comparators and mixed mean-based and median-based reporting; thus, the effect could not be quantified reliably. With respect to muscle strength, FIM and pain, the evidence is based on one or two small studies, one of which, Middaugh et al. (12), reported only within-group comparisons; thus, no between-group effect could be estimated for pain. For MAS, the outcome data type was inconsistent across studies, and the three-arm trial by Gong and Zhang (17) suggested that EMG-BF may not confer additional benefit over an active alternative for spasticity.

The most important limitation of this evidence base is the heterogeneity of the control conditions. Although the eligibility criteria required conventional rehabilitation as the comparator, the actual comparator arms ranged from conventional rehabilitation alone to several different active alternatives, including medium-frequency electrotherapy, core-stability training and suspension exercise. This means that the available studies estimate the advantage of EMG-BF over a heterogeneous blend of therapies rather than over no treatment or usual care alone, and it is a principal reason why quantitative pooling was not appropriate. A second important limitation is the reliability of the primary data: During the revision, we re-extracted all the outcomes and corrected several coding problems that affected the earlier analysis, including a reversal of treatment and comparator groups in one study, the treatment of ordinal MAS grades and of median (IQR) data as the means (SD), and an implausible muscle strength value identical to another study’s sEMG value. These problems, together with the near-universal absence of allocation concealment and blinding and the small sample sizes, carry a genuine risk of bias and limit the internal validity of any quantitative estimate.

EMG-BF is also useful in the context of other adjunctive neurorehabilitation technologies for SCI. Functional electrical stimulation (FES), repetitive transcranial magnetic stimulation (rTMS) and robot-assisted gait training have been evaluated in randomized trials and pooled analyses, with effect sizes broadly comparable in magnitude to those reported here for EMG-BF (36–38). Direct quantitative comparison is precluded by differences in populations, outcome measures and comparator conditions, but the EMG-BF appears to belong to a family of adjunctive, low-risk interventions that produce modest-to-moderate gains when added to conventional rehabilitation. Whether EMG-BF is superior, equivalent or inferior to these alternatives remains an open question requiring head-to-head trials, and our findings therefore support the use of EMG-BF as one available option rather than a clearly preferred therapy.

Publication bias could not be formally assessed for any outcome because every outcome included fewer than 10 studies. This limitation is inherent to a field dominated by small, single-center trials and should be weighed when interpreting the results. This review has several further limitations: most included studies were conducted in a single country (China), and several narrowly selected subgroups (e.g., incomplete thoracolumbar injuries, lower-limb spasticity, or shoulder pain in manual wheelchair users) were included, limiting generalisability; intervention parameters—treatment duration, session frequency, intensity, target muscles and device type—varied substantially across studies; and electromyographic signals are inherently susceptible to electrode placement and signal-processing artifacts. Collectively, these shortcomings mean that the present findings should be regarded as hypothesis-generating and supportive of — but not definitive for — the routine clinical use of EMG-BF, pending larger, multicenter, adequately concealed RCTs with standardized comparators and outcomes.

## 5 Conclusion

Low- to very-low-certainty evidence suggests that EMG-BF may improve sEMG signals, lower-limb muscle strength, functional independence, ASIA motor scores, spasticity and balance in patients with SCI. Because every included study was judged to be at some risk of bias, most outcomes were based on only one to four small studies, and the comparator conditions were highly heterogeneous; these findings should be interpreted with caution rather than as established benefits. EMG-BF is noninvasive and easy to administer and may be considered a promising adjunct to conventional rehabilitation; however, its effectiveness remains to be confirmed by higher-certainty evidence. Future research should include large, multicenter randomized controlled trials with adequate allocation concealment, blinding of outcome assessors, standardized comparators and prospective registration and should further develop closed-loop intelligent feedback systems integrating electromyographic signals with electroencephalography (EEG) and functional near-infrared spectroscopy (fNIRS) to determine optimal treatment parameters and intervention cycles for EMG-BF.

## Data Availability

All the data generated or analyzed during this study are included in this published article and its Supplementary files.
