# Supplementary material for: Electromyography biofeedback therapy for spinal cord injury rehabilitation: a systematic review
Source: Front Neurol. 2026 Oct 13;17:1885605. doi: 10.3389/fneur.2026.1885605 (PMC13710046; doi:10.3389/fneur.2026.1885605)
Supplement: Supplementary file 1 [file Supplementary_file_1.docx]

**Supplementary Table S1. Domain-level risk-of-bias judgements and the supporting evidence for each included study.**

The 10 randomised trials were assessed with the Cochrane risk-of-bias tool for randomised trials (RoB 2) across five domains. Two reviewers assessed each study independently and resolved disagreements by discussion. Under RoB 2, the overall judgement is “Low” only when every domain is low, “High” when any domain is high, and “Some concerns” otherwise.

Quotations are reproduced verbatim from the primary report. Where the primary report is in Chinese, a faithful English translation is given and marked “[translated from Chinese]”. “Not reported” means the item was searched for and is absent from the primary report; it is not an inference.

**Panel A. Randomised controlled trials (RoB 2, n = 10)**

*Domains: D1 randomisation process; D2 deviations from intended interventions; D3 missing outcome data; D4 measurement of the outcome; D5 selection of the reported result.*

| **Study** | **Domain** | **Judgement** | **Support for judgement** |
| --- | --- | --- | --- |
| **Govil & Noohu 2013 (13)**  *Govil K, Noohu MM. Effect of EMG biofeedback training of gluteus maximus muscle on gait parameters in incomplete spinal cord injury. NeuroRehabilitation. 2013;33(1):147–152. DOI: 10.3233/NRE-130939.*  Design: Parallel-group RCT, pretest–posttest design | D1 Randomisation process | Some concerns | "30 incomplete spinal cord injured (ISCI) patients were included and randomly assigned to two groups." Randomisation method and allocation concealment not reported; baseline ASIA score Mode differed between groups (D vs C). |
|  | D2 Deviations from intended interventions | Some concerns | "EMG Biofeedback was given specifically over gluteus maximus muscle along with traditional rehabilitation and gait training to Group 1 … Group 2 received traditional rehabilitation and gait training." Blinding of participants, personnel and outcome assessors not reported. |
|  | D3 Missing outcome data | Some concerns | "A total of 30 subjects with ISCI were recruited for the study …" No dropouts, losses or per-group analysis n reported, so completeness of outcome data cannot be verified. |
|  | D4 Measurement of the outcome | Low | "The steplength was calculated as linear distance between two successive heel contacts …" and "The integrated EMG amplitude (micro-volts) during the attempted hip extension was recorded." Objective instrument-based outcomes measured identically in both groups. |
|  | D5 Selection of the reported result | Some concerns | Not reported. No trial registration or prospective protocol; only ethics approval reported, so selective reporting cannot be excluded. |
|  | **Overall** | **Some concerns** | Four domains at Some concerns and none at High; decisive concerns are the unreported randomisation method and allocation concealment (D1) and absence of any prospective registration (D5). |
| **Guo et al. 2021 (**18**)**  *Guo Y, Gao F, Li J, Yang M, Li J, Yang D, Du L. Effect of electromyographic biofeedback training on motor function of quadriceps femoris in patients with incomplete spinal cord injury: A randomized controlled trial. *NeuroRehabilitation* 2021;48(3):345–351. DOI: 10.3233/NRE-201647*  Design: Parallel-group randomised controlled trial | D1 Randomisation process | Low | "A block randomization with random block sizes of 2 or 4 was performed by a specialist who was involved neither in the intervention nor in the assessments." Sealed, consecutively numbered envelopes; "No statistically significant differences … in baseline measures between the two groups." |
|  | D2 Deviations from intended interventions | Some concerns | Blinding of participants and personnel: Not reported. "Intervention group received EMG BF along with conventional physical therapy, while control group only received conventional physical therapy." Whether analysis was ITT is not stated. |
|  | D3 Missing outcome data | Some concerns | "40 patients were recruited and allocated to either intervention group or control group using a random sequence." vs "33 incomplete SCI patients … in this trail finally." 7 (17.5%) unaccounted for; no dropout reasons reported. |
|  | D4 Measurement of the outcome | Some concerns | "Surface electromyograph (sEMG), muscle strength and thigh circumference size were assessed… Activities of daily living (ADL) was evaluated by Modified Barthel Index (MBI)." Outcome assessor blinding: Not reported; muscle strength and MBI are assessor-dependent. |
|  | D5 Selection of the reported result | Low | "The clinical trial was registered in the Chinese Clinical Trial Registry (no. ChiCTR1800018891…)." Prospectively registered; all four prespecified outcomes reported in Results/Table 2. |
|  | **Overall** | **Some concerns** | D1 and D5 are Low, D2–D4 are Some concerns; overall equals the highest domain risk. |
| **Middaugh et al. 2013 (12)**  *Middaugh S, Thomas KJ, Smith AR, McFall TL, Klingmueller J. EMG Biofeedback and Exercise for Treatment of Cervical and Shoulder Pain in Individuals with a Spinal Cord Injury: A Pilot Study. *Top Spinal Cord Inj Rehabil.* 2013;19(4):311–323. doi:10.1310/sci1904-311*  Design: Prospective parallel-group pilot RCT | D1 Randomisation process | Some concerns | "After baseline 2, participants were randomly assigned to 1 of 2..." — randomisation method and allocation concealment not reported. |
|  | D2 Deviations from intended interventions | Some concerns | "all study participants, in both groups, completed the 10-week study protocol." Participants knew their group; outcome was self-reported. |
|  | D3 Missing outcome data | Low | "Fourteen of 15 participants returned to the study site... One participant (group A) was interviewed by phone and completed the WUSPI by mail." No posttest loss; follow-up value single-imputed with group mean. |
|  | D4 Measurement of the outcome | Some concerns | "Baseline and posttest data were collected by an evaluator who was blind to group assignment." Self-report pain scale; participants unblinded. |
|  | D5 Selection of the reported result | Some concerns | "Follow-up was not part of the planned prospective study but was an exploratory addition." No registration or prespecified protocol. |
|  | **Overall** | **Some concerns** | Small pilot RCT with unblinded self-reported outcome, unreported randomisation/concealment, and exploratory post-hoc follow-up; no domain high. |
| **He H et al., 2011(11)**  *HE Huai (何怀). 肌电生物反馈训练对脊髓损伤患者心理功能及运动功能的影响 [Effects of electromyographic biofeedback training on psychological and motor function in patients with spinal cord injury]. 中华物理医学与康复杂志 [Chinese Journal of Physical Medicine and Rehabilitation], 2011, 33(10): 762–765. DOI: 10.3760/cma.j.issn.0254-1424.2011.010.011*  Design: Parallel-group RCT (random number table) | D1 Randomisation process | Some concerns | "A total of 70 SCI patients were selected and divided into a treatment group and a control group using a random number table" [translated from Chinese]. Allocation concealment: Not reported. |
|  | D2 Deviations from intended interventions | Some concerns | Not reported. Blinding of patients and treating therapists, and any deviations from intended interventions, are not described. |
|  | D3 Missing outcome data | Low | "A total of 70 SCI patients hospitalised … between January 2008 and December 2009 were selected", divided 35 per group; both groups analysed as 35. Dropouts/losses: Not reported. |
|  | D4 Measurement of the outcome | Some concerns | "The Rosenberg Self-Esteem Scale (RSES) was used to assess the psychological state of the two groups before and after treatment" [translated from Chinese]; outcome assessor blinding: Not reported. |
|  | D5 Selection of the reported result | Some concerns | Not reported. No prospective registration or pre-specified analysis plan; all outcomes listed in Methods are reported in Results. |
|  | **Overall** | **Some concerns** | Allocation concealment and participant, therapist and outcome-assessor blinding were all unreported, with no prospective registration; only D3 (missing outcome data) was judged Low. |
| **Shi et al. 2014 (14)**  *石秀秀, 唐金树, 秦江, 等. 肌电生物反馈疗法配合踝足支具治疗脊髓损伤患者术后踝背屈功能障碍的疗效. 中国骨与关节杂志 [Chinese Journal of Bone and Joint], 2014, 3(9): 661-664.*  Design: Parallel-group RCT (claimed randomisation) | D1 Randomisation process | Some concerns | "They were randomly divided into observation group ( n=15 ) and control group ( n=15 )" (English abstract); Chinese methods record only "all cases were divided into observation and control groups, 15 each" [translated from Chinese] with no random-sequence method. Allocation concealment: Not reported. |
|  | D2 Deviations from intended interventions | Some concerns | Blinding of participants and personnel: Not reported. "对照组不进行肌电生物反馈电刺激疗法，只进行基础训练" = "the control group did not undergo EMG biofeedback electrical stimulation and received only basic training" [translated from Chinese] — no sham intervention. All treatment by the same therapy team. |
|  | D3 Missing outcome data | Low | 30 cases entered analysis, both groups n=15 with complete before/after data in Tables 1-2; "经过 3 个月治疗，两组患者的 sEMG 信号及肌力均有提高" = "after 3 months of treatment, sEMG signals and muscle strength improved in both groups" [translated from Chinese]; no dropouts reported. |
|  | D4 Measurement of the outcome | Some concerns | sEMG signal (objective instrument) plus manual Lovett muscle-strength grading (subjective). "由康复医师、物理治疗师共同完成对患者的疗效评定" = "outcome assessment was completed jointly by rehabilitation physicians and physical therapists" [translated from Chinese], unblinded and the same team as treatment. |
|  | D5 Selection of the reported result | Some concerns | No prospective registration or protocol: Not reported. Both prespecified outcomes (sEMG, muscle strength) were reported; selective reporting cannot be excluded. |
|  | **Overall** | **Some concerns** | D1, D2, D4, D5 are Some concerns and D3 is Low with no High domain — randomisation method and allocation concealment unreported, no blinding, and no protocol or registration. |
| **Gong QL et al. 2021(17)**  *龚巧鹭, 张建梅. 肌电生物反馈及悬吊运动训练对脊髓损伤后下肢痉挛患者步行功能的影响[J]. 中国医药导报 [China Medical Herald], 2021, 18(2): 110-113. DOI:10.20047/j.issn1673-7210.2021.02.028*  Design: Randomised controlled trial (three-group parallel, single-centre) | D1 Randomisation process | Some concerns | "依据随机数字表法分为 A组、B组和C组，各40例" ("divided into groups A, B and C by the random number table method, 40 cases each") [translated from Chinese]; allocation concealment: Not reported. |
|  | D2 Deviations from intended interventions | Some concerns | No blinding of patients or therapists reported; "均干预 2 个月" ("all were treated for 2 months") [translated from Chinese]; intention-to-treat analysis: Not reported. |
|  | D3 Missing outcome data | Low | All outcome tables report n=40 per group before and after intervention ("组别 例数：A组 40 / B组 40 / C组 40"); no dropout or loss to follow-up reported. |
|  | D4 Measurement of the outcome | Some concerns | Outcomes are clinical rating scales (FAC, MAS, CSS, BBS, Fugl-Meyer) with subjective components; outcome assessor blinding: Not reported. |
|  | D5 Selection of the reported result | Some concerns | Methods list FAC, MAS, CSS, BBS and Fugl-Meyer; results report all five consistently; prospective trial registration or protocol: Not reported. |
|  | **Overall** | **Some concerns** | Four of five domains are at "some concerns" and none is high, driven mainly by un-reported allocation concealment and blinding. |
| **Yang S et al. 2023 (19)**  *杨升, 吴博中. 肌电生物反馈电刺激改善脊髓损伤患者下肢功能及步行能力的研究. 现代医学与健康研究 [Modern Medicine and Health Research], 2023, 7(24).*  Design: Parallel-group RCT (random number table method) | D1 Randomisation process | Some concerns | "divided into two groups by the random number table method" [translated from Chinese]; allocation concealment: Not reported; baseline characteristics comparable (P>0.05). |
|  | D2 Deviations from intended interventions | Some concerns | Not reported (no statement on blinding of participants/personnel, deviations from intervention, or intention-to-treat analysis). |
|  | D3 Missing outcome data | Low | "control group (21 cases) … observation group (21 cases)" [translated from Chinese]; all result tables show 21/21 at every time point; no dropout or loss to follow-up reported. |
|  | D4 Measurement of the outcome | Some concerns | Assessor blinding: Not reported; outcomes include subjective scales (LMES, MAS, BBS, WCS, NBDS) judged Some concerns, while objective laboratory markers (PDGF/SP/VIP) are unaffected. |
|  | D5 Selection of the reported result | Some concerns | Not reported (no trial registration number or protocol); all outcomes listed in Methods are reported in Results. |
|  | **Overall** | **Some concerns** | Allocation concealment, blinding, and prospective registration were not reported, despite adequate random sequence generation. |
| **Hu et al.2016(15)**  *胡可慧, 常有军, 杨廷彦, 张逸, 田野, 李阳安. 肌电生物反馈联合康复训练对脊髓损伤患者运动功能及功能独立性的影响. 中国康复 [Chinese Journal of Rehabilitation], 2016, 31(6): 450-452. DOI: 10.3870/zgkf.2016.06.013*  Design: Claimed parallel-group RCT | D1 Randomisation process | Some concerns | "All cases were randomly divided into 2 groups of 30 cases each." [translated from Chinese] (Methods). No random-sequence generation method (no random number table, computer, or lots) and no allocation concealment reported. "There was no statistically significant difference in general data between the two groups." [translated from Chinese]. |
|  | D2 Deviations from intended interventions | Some concerns | "Both groups received routine rehabilitation training; the experimental group additionally received EMG biofeedback therapy." [translated from Chinese]. No sham/placebo control; blinding (patients/personnel) Not reported. |
|  | D3 Missing outcome data | Low | "Sixty cases of incompletely thoracic-lumbar SCI were randomly divided into experimental group and control group, 30 cases in each group." [from English abstract]. n=30/30 complete at all time points (Tables 1, 2); no dropout/loss to follow-up reported. |
|  | D4 Measurement of the outcome | Some concerns | sEMG measured with WOND2000F2 instrument (objective); ASIA motor score and FIM scales carry subjective components; outcome assessor blinding Not reported. |
|  | D5 Selection of the reported result | Some concerns | No prospective registration, no study protocol, no ethics approval number (Not reported); all three pre-specified outcomes reported at 8 and 12 weeks, but cannot verify against any protocol. |
|  | **Overall** | **Some concerns** | Four domains Some concerns (D1, D2, D4, D5), D3 Low, none High; driven by unreported randomisation method and allocation concealment, absence of blinding, and no protocol/registration. |
| **Chen et al,2024(20)**  *陈晓, 张晗. 肌电生物反馈联合非稳定平面核心稳定训练治疗脊髓损伤患者观察[J]. 中国卫生产业 [China Health Industry], 2024, 42(20): 66-69.*  Design: Parallel-group RCT (random number table allocation) | D1 Randomisation process | Some concerns | "divided into a control group and an observation group using the random number table method, 38 cases in each group" [translated from Chinese]; baseline characteristics comparable (P>0.05). Allocation concealment: Not reported. |
|  | D2 Deviations from intended interventions | Some concerns | Physical rehabilitation vs combined EMG biofeedback is hard to blind; patient and practitioner blinding and any deviations or ITT analysis: Not reported. |
|  | D3 Missing outcome data | Low | "no serious complications occurred in either group during the intervention" [translated from Chinese]; all outcome tables show 38/38 per group post-intervention, no missing data. |
|  | D4 Measurement of the outcome | Some concerns | "before and after the intervention, 3 nursing staff assessed the patients' walking ability and took the average" [translated from Chinese]; outcome assessor blinding: Not reported. |
|  | D5 Selection of the reported result | Some concerns | Six outcomes listed in Methods (FAC, FMA, BBS, BI, MAS, complications) are all reported (2.1–2.4); prospective registration/protocol: Not reported. |
|  | **Overall** | **Some concerns** | Allocation concealment, all blinding (patients/practitioners/assessors), and prospective registration were not reported, while outcome data were complete. |
| **Xie et al,2019(16)**  *解姗姗, 马仁华. 肌电生物反馈疗法治疗脊髓损伤下肢痉挛患者的效果观察. 中国民康医学 [Medical Journal of Chinese People's Health], 2019, 31(4): 90-91.*  Design: Randomised controlled trial (two parallel groups) | D1 Randomisation process | Some concerns | Random sequence: "divided into two groups by the random number table method, 27 cases each" [translated from Chinese]. Allocation concealment: Not reported. Baseline: "ASIA grade, age, sex, injury level ... differences not statistically significant (P>0.05)" [translated from Chinese]. |
|  | D2 Deviations from intended interventions | Some concerns | Blinding of participants/personnel: Not reported (rehabilitation intervention inherently hard to blind). Deviations or co-interventions: Not reported. Analysis n=27 per group throughout, no dropouts reported. |
|  | D3 Missing outcome data | Low | All outcome tables labelled n=27 per group, consistent before and after treatment; no dropout or loss to follow-up described in the full text. |
|  | D4 Measurement of the outcome | Some concerns | Blinding of outcome assessors: Not reported. Outcomes include the modified Ashworth scale (assessor-dependent) and GQOL-74 quality-of-life questionnaire (patient self-report); "patients and family gave informed consent" [translated from Chinese] relates to consent, not blinding. |
|  | D5 Selection of the reported result | Some concerns | No prospective protocol or registration reported. The outcomes listed in Methods (spasticity, range of motion, quality of life) are all reported in the results; no evidence of selective reporting. |
|  | **Overall** | **Some concerns** | No domain judged High; D3 is Low and the remaining four domains are Some concerns. |
