# Supplementary material for: Electromyography biofeedback therapy for spinal cord injury rehabilitation: a systematic review
Source: Front Neurol. 2026 Oct 13;17:1885605. doi: 10.3389/fneur.2026.1885605 (PMC13710046; doi:10.3389/fneur.2026.1885605)
Supplement: Supplementary file 2 [file Supplementary_file_2.docx]

**Supplementary**

**Search strategy**

**Catalogue**

•Research question

•Retrieve database

•Search strategy

o[PubMed](" \l "database-pubmed)

o[Embase](" \l "database-embase)

o[Web of Science](" \l "database-web-of-science)

o[Cochrane Library](" \l "database-cochrane-library)

o[China National Knowledge Infrastructure (CNKI)](" \l "database-cnki)

oWanfang Database

**Research question**

PICO framework

**P (Population)**: Patients with spinal cord injury

**I (Intervention)**: Electromyographic biofeedback therapy

**C (Comparison)**: Conventional rehabilitation therapy/placebo/no treatment

**O (Outcome)**: Rehabilitation outcome indicators such as motor function recovery, activities of daily living ability, gait parameters, and muscle strength

Retrieve database

[Meta-analysis of the impact of electrobiofeedback therapy on the rehabilitation outcomes of patients with spinal cord injury:](#_Toc2025207684)

[Search strategy	1](#_Toc2025207684)

[Catalogue	1](#_Toc49740038)

[Research question	1](#_Toc609679983)

[Retrieve database	1](#_Toc942709235)

[Search strategy	2](#_Toc2127248726)

[Database: PubMed <inception to 2025>	2](#_Toc1361582626)

[Database: Embase <1974 to 2025>	4](#_Toc533452750)

[Database: Web of Science <1965 to 2025>	5](#_Toc2143626672)

[Database: Cochrane Library	6](#_Toc1747814232)

[Database: China National Knowledge Infrastructure(CNKI)	7](#_Toc84989911)

[Database: Wanfang Database	7](#_Toc348808922)

Search strategy

Database: PubMed <inception to 2025>

Search strategy:

| Retrieval number | **Search query** |
| --- | --- |
| 1 | "Spinal Cord Injuries"[MeSH Terms] |
| 2 | (spinal cord injur*[Title/Abstract]) OR (spinal cord trauma*[Title/Abstract]) OR (spinal cord lesion*[Title/Abstract]) OR (spinal cord transection*[Title/Abstract]) OR (spinal cord laceration*[Title/Abstract]) OR (spinal cord contusion*[Title/Abstract]) OR (spinal cord hemisection*[Title/Abstract]) OR (traumatic myelopath*[Title/Abstract]) OR (spinal cord damage*[Title/Abstract]) OR (spinal cord dysfunction*[Title/Abstract]) OR (SCI[Title/Abstract]) OR (traumatic paraplegia[Title/Abstract]) OR (traumatic tetraplegia[Title/Abstract]) OR (traumatic quadriplegia[Title/Abstract]) |
| 3 | 1 OR 2 |
| 4 | "Biofeedback, Psychology"[MeSH Terms] OR "Feedback, Sensory"[MeSH Terms] OR "Electromyography"[MeSH Terms] |
| 5 | (biofeedback[Title/Abstract]) OR (bio-feedback[Title/Abstract]) OR (EMG biofeedback[Title/Abstract]) OR (electromyographic biofeedback[Title/Abstract]) OR (myofeedback[Title/Abstract]) OR (neurofeedback[Title/Abstract]) OR (electromyogram biofeedback[Title/Abstract]) OR (sensory feedback[Title/Abstract]) OR (proprioceptive feedback[Title/Abstract]) OR (visual feedback[Title/Abstract]) OR (auditory feedback[Title/Abstract]) |
| 6 | 4 OR 5 |
| 7 | "Rehabilitation"[MeSH Terms] OR "Recovery of Function"[MeSH Terms] OR "Treatment Outcome"[MeSH Terms] OR "Motor Skills"[MeSH Terms] |
| 8 | (rehabilitation[Title/Abstract]) OR (functional recovery[Title/Abstract]) OR (motor recovery[Title/Abstract]) OR (motor function[Title/Abstract]) OR (functional improvement[Title/Abstract]) OR (physical therapy[Title/Abstract]) OR (physiotherapy[Title/Abstract]) OR (motor control[Title/Abstract]) OR (muscle strength[Title/Abstract]) OR (gait[Title/Abstract]) OR (walking ability[Title/Abstract]) OR (activities of daily living[Title/Abstract]) OR (ADL[Title/Abstract]) OR (mobility[Title/Abstract]) OR (balance[Title/Abstract]) OR (spasticity[Title/Abstract]) |
| 9 | 7 OR 8 |
| 10 | "Randomized Controlled Trial"[Publication Type] OR "Controlled Clinical Trial"[Publication Type] OR "Clinical Trial"[Publication Type] OR "Meta-Analysis"[Publication Type] |
| 11 | (random*[Title/Abstract]) OR (placebo[Title/Abstract]) OR (trial[Title/Abstract]) OR (groups[Title/Abstract]) OR (clinical trial*[Title/Abstract]) OR (controlled clinical trial[Title/Abstract]) OR (randomized[Title/Abstract]) OR (randomised[Title/Abstract]) |
| 12 | 10 OR 11 |
| 13 | 3 AND 6 AND 9 AND 12 |
| 14 | (animals[MeSH Terms]) NOT (humans[MeSH Terms]) |
| 15 | 13 NOT 14 |

Database: Embase <1974 to 2025>

Search strategy:

1. 'spinal cord injury'/exp
2. (spinal NEAR/2 cord NEAR/2 (injur* OR trauma* OR lesion* OR transection* OR laceration* OR contusion* OR hemisection* OR damage* OR dysfunction*)),ti OR 'sci',ti OR (traumatic NEAR/2 (paraplegia OR tetraplegia OR quadriplegia OR myelopath*)),ti

3.1 OR 2

3. 'feedback system'/exp OR 'biofeedback'/exp OR 'electromyography'/exp
4. biofeedback,ti OR 'bio-feedback',ti OR (emg NEAR/2 biofeedback),ti OR (electromyographic NEAR/2 biofeedback),ti OR myofeedback,ti OR neurofeedback,ti OR (electromyogram NEAR/2 biofeedback),ti OR (sensory NEAR/2 feedback),ti OR (proprioceptive NEAR/2 feedback),ti OR (visual NEAR/2 feedback),ti OR (auditory NEAR/2 feedback),ti

6.4 OR 5

5. 'rehabilitation'/exp OR 'convalescence'/exp OR 'treatment outcome'/exp OR 'motor performance'/exp
6. rehabilitation,ti OR (functional NEAR/2 recovery),ti OR (motor NEAR/2 recovery),ti OR (motor NEAR/2 function),ti OR (functional NEAR/2 improvement),ti OR 'physical therapy',ti OR physiotherapy,ti OR (motor NEAR/2 control),ti OR (muscle NEAR/2 strength),ti OR gait,ti OR (walking NEAR/2 ability),ti OR (activities NEAR/2 'daily living'),ti OR adl,ti OR mobility,ti OR balance,ti OR spasticity,ti

9.7 OR 8

7. 'randomized controlled trial'/exp OR 'controlled clinical trial'/exp OR 'clinical trial'/exp OR 'meta analysis'/exp
8. random*,ti OR placebo,ti OR trial,ti OR groups,ti OR (clinical NEXT/1 trial*),ti OR (controlled NEXT/1 clinical NEXT/1 trial),ti OR randomized,ti OR randomised,ti

12.10 OR 11

13.3 AND 6 AND 9 AND 12

9. 'animal'/exp NOT 'human'/exp

15.13 NOT 14

Database: Web of Science <1965 to 2025>

Search strategy:

:

| Retrieval number | Search query |
| --- | --- |
| 1 | TOPIC: (spinal cord injur* OR spinal cord trauma* OR spinal cord lesion* OR spinal cord transection* OR spinal cord laceration* OR spinal cord contusion* OR spinal cord hemisection* OR traumatic myelopath* OR spinal cord damage* OR spinal cord dysfunction* OR SCI OR traumatic paraplegia OR traumatic tetraplegia OR traumatic quadriplegia) |
| 2 | TOPIC: (biofeedback OR bio-feedback OR EMG biofeedback OR electromyographic biofeedback OR myofeedback OR neurofeedback OR electromyogram biofeedback OR sensory feedback OR proprioceptive feedback OR visual feedback OR auditory feedback) |
| 3 | TOPIC: (rehabilitation OR functional recovery OR motor recovery OR motor function OR functional improvement OR physical therapy OR physiotherapy OR motor control OR muscle strength OR gait OR walking ability OR activities of daily living OR ADL OR mobility OR balance OR spasticity) |
| 4 | TOPIC: (random* OR placebo OR trial OR groups OR clinical trial* OR controlled clinical trial OR randomized OR randomised) |
| 5 | 1 AND 2 AND 3 AND 4 |

Database: Cochrane Library

Search strategy:

1 MeSH descriptor: [Spinal Cord Injuries] explode all trees 2 (spinal cord injur* OR spinal cord trauma* OR spinal cord lesion* OR spinal cord transection* OR spinal cord laceration* OR spinal cord contusion* OR spinal cord hemisection* OR traumatic myelopath* OR spinal cord damage* OR spinal cord dysfunction* OR SCI OR traumatic paraplegia OR traumatic tetraplegia OR traumatic quadriplegia)

,ab,kw 3 1 OR 2 4 MeSH descriptor: [Biofeedback, Psychology] explode all trees 5 MeSH descriptor: [Feedback, Sensory] explode all trees 6 MeSH descriptor: [Electromyography] explode all trees 7 (biofeedback OR bio-feedback OR EMG biofeedback OR electromyographic biofeedback OR myofeedback OR neurofeedback OR electromyogram biofeedback OR sensory feedback OR proprioceptive feedback OR visual feedback OR auditory feedback),ab,kw 8 4 OR 5 OR 6 OR 7 9 MeSH descriptor: [Rehabilitation] explode all trees 10 MeSH descriptor: [Recovery of Function] explode all trees 11 MeSH descriptor: [Treatment Outcome] explode all trees 12 MeSH descriptor: [Motor Skills] explode all trees 13 (rehabilitation OR functional recovery OR motor recovery OR motor function OR functional improvement OR physical therapy OR physiotherapy OR motor control OR muscle strength OR gait OR walking ability OR activities of daily living OR ADL OR mobility OR balance OR spasticity),ab,kw 14 9 OR 10 OR 11 OR 12 OR 13 15 3 AND 8 AND 14

Database: 中国知网(CNKI)

**检索号**:

| **检索号** | **检索式** |
| --- | --- |
| 1 | 主题:(脊髓损伤 OR 脊髓创伤 OR 脊髓病变 OR 脊髓切断 OR 脊髓横断 OR 脊髓挫伤 OR SCI OR 外伤性截瘫 OR 外伤性四肢瘫 OR 外伤性脊髓病变) |
| 2 | 主题:(肌电生物反馈 OR 生物反馈 OR 肌电图生物反馈 OR 神经反馈 OR 感觉反馈 OR 本体感觉反馈 OR 视觉反馈 OR 听觉反馈) |
| 3 | 主题:(康复 OR 功能恢复 OR 运动功能恢复 OR 运动控制 OR 肌力 OR 步态 OR 行走能力 OR 日常生活活动能力 OR ADL OR 活动度 OR 平衡 OR 痉挛) |
| 4 | 主题:(随机 OR 对照 OR 试验 OR 临床试验 OR 随机对照试验 OR 随机分配 OR 安慰剂) |
| 5 | 1 AND 2 AND 3 AND 4 |

Database: 万方数据库

搜索策略:

| **检索号** | **检索式** |
| --- | --- |
| 1 | 主题:(脊髓损伤 OR 脊髓创伤 OR 脊髓病变 OR 脊髓切断 OR 脊髓横断 OR 脊髓挫伤 OR SCI OR 外伤性截瘫 OR 外伤性四肢瘫 OR 外伤性脊髓病变) |
| 2 | 主题:(肌电生物反馈 OR 生物反馈 OR 肌电图生物反馈 OR 神经反馈 OR 感觉反馈 OR 本体感觉反馈 OR 视觉反馈 OR 听觉反馈) |
| 3 | 主题:(康复 OR 功能恢复 OR 运动功能恢复 OR 运动控制 OR 肌力 OR 步态 OR 行走能力 OR 日常生活活动能力 OR ADL OR 活动度 OR 平衡 OR 痉挛) |
| 4 | 主题:(随机 OR 对照 OR 试验 OR 临床试验 OR 随机对照试验 OR 随机分配 OR 安慰剂) |
| 5 | 1 AND 2 AND 3 AND 4 |
